# Acetylcholine Delays Atrial Activation to Facilitate Atrial Fibrillation

**DOI:** 10.3389/fphys.2019.01105

**Published:** 2019-09-04

**Authors:** Jason D. Bayer, Bastiaan J. Boukens, Sébastien P. J. Krul, Caroline H. Roney, Antoine H. G. Driessen, Wouter R. Berger, Nicoline W. E. van den Berg, Arie O. Verkerk, Edward J. Vigmond, Ruben Coronel, Joris R. de Groot

**Affiliations:** ^1^Electrophysiology and Heart Modeling Institute (IHU-LIRYC), Bordeaux University Foundation, Bordeaux, France; ^2^Institute of Mathematics of Bordeaux (U5251), University of Bordeaux, Bordeaux, France; ^3^Department of Medical Biology, Academic Medical Center, Amsterdam, Netherlands; ^4^Department of Cardiology, Academic Medical Center, Amsterdam, Netherlands; ^5^Division of Imaging Sciences and Bioengineering, King’s College London, London, United Kingdom; ^6^Department of Cardiology, Heart Center, OLVG, Amsterdam, Netherlands; ^7^Department of Experimental Cardiology, Academic Medical Center, Amsterdam, Netherlands

**Keywords:** atria, fibrillation, acetylcholine, conduction, fibrosis, computational modeling

## Abstract

**Background:**

Acetylcholine (ACh) shortens action potential duration (APD) in human atria. APD shortening facilitates atrial fibrillation (AF) by reducing the wavelength for reentry. However, the influence of ACh on electrical conduction in human atria and its contribution to AF are unclear, particularly when combined with impaired conduction from interstitial fibrosis.

**Objective:**

To investigate the effect of ACh on human atrial conduction and its role in AF with computational, experimental, and clinical approaches.

**Methods:**

S1S2 pacing (S1 = 600 ms and S2 = variable cycle lengths) was applied to the following human AF computer models: a left atrial appendage (LAA) myocyte to quantify the effects of ACh on APD, maximum upstroke velocity (V_*max*_), and resting membrane potential (RMP); a monolayer of LAA myocytes to quantify the effects of ACh on conduction; and 3) an intact left atrium (LA) to determine the effects of ACh on arrhythmogenicity. Heterogeneous ACh and interstitial fibrosis were applied to the monolayer and LA models. To corroborate the simulations, APD and RMP from isolated human atrial myocytes were recorded before and after 0.1 μM ACh. At the tissue level, LAAs from AF patients were optically mapped *ex vivo* using Di-4-ANEPPS. The difference in total activation time (AT) was determined between AT initially recorded with S1 pacing, and AT recorded during subsequent S1 pacing without (*n* = 6) or with (*n* = 7) 100 μM ACh.

**Results:**

In LAA myocyte simulations, S1 pacing with 0.1 μM ACh shortened APD by 41 ms, hyperpolarized RMP by 7 mV, and increased V_*max*_ by 27 mV/ms. In human atrial myocytes, 0.1 μM ACh shortened APD by 48 ms, hyperpolarized RMP by 3 mV, and increased V_*max*_ by 6 mV/ms. In LAA monolayer simulations, S1 pacing with ACh hyperpolarized RMP to delay total AT by 32 ms without and 35 ms with fibrosis. This led to unidirectional conduction block and sustained reentry in fibrotic LA with heterogeneous ACh during S2 pacing. In AF patient LAAs, S1 pacing with ACh increased total AT from 39.3 ± 26 ms to 71.4 ± 31.2 ms (*p* = 0.036) compared to no change without ACh (56.7 ± 29.3 ms to 50.0 ± 21.9 ms, *p* = 0.140).

**Conclusion:**

In fibrotic atria with heterogeneous parasympathetic activation, ACh facilitates AF by shortening APD and slowing conduction to promote unidirectional conduction block and reentry.

## Introduction

The parasympathetic neurotransmitter acetylcholine (ACh) activates the outward potassium current I_*KACh*_ (Krapivinsky et al., 1995). In atrial myocytes, this substantially shortens action potential duration (APD) (Zaza et al., 1995). Consequently, APD shortening facilitates the onset and maintenance of atrial fibrillation (AF) by reducing the wavelength for reentry (Smeets et al., 1986), defined as APD^∗^conduction velocity (CV). In other words, as the wavelength for reentry shortens, AF susceptibility increases due to less atrial tissue needed to initiate and harbor sustained reentrant circuits.

The effect of ACh on potassium currents in atrial myocytes has been studied for the past 65 years (Acierno et al., 1952; Johnson and Robertson, 1957, 1958; Heidbuchel et al., 1987). Building upon these studies, there is convincing evidence linking shortened APD from elevated ACh to AF in patients (Krummen et al., 2012) and animal models (Roney et al., 2018), as well as an abundance of computational studies studying its effects on sinoatrial node function (Michaels et al., 1984; Egan and Noble, 1987; Dexter et al., 1989; Moss et al., 2018). However, literature for the effect ACh on conduction with respect to AF in patients is limited.

In large mammals, conflicting studies suggest ACh has no effect (Schuessler et al., 1991) or slows atrial conduction (Lin et al., 2007), with the latter suggested to promote unidirectional conduction block and reentrant arrhythmias. Altered conduction may result from ACh reducing tissue excitability via hyperpolarization of the resting membrane potential (RMP) (Pott, 1979; Molina et al., 2007; Verkerk et al., 2012), which may be enhanced by impaired conduction from heterogeneous interstitial fibrosis (Koduri et al., 2012; Krul S.P. et al., 2015). However, the effect of ACh on atrial conduction in humans with structural abnormalities and its role in AF have not been systematically studied.

Fortunately, the link between AF and fibrosis has been systematically studied. In AF patients, late gadolinium-enhanced cardiac magnetic resonance imaging shows the number and location of AF reentrant drivers to correlate with fibrosis density (Cochet et al., 2018). Furthermore, slow conduction from increased total fibrosis would reduce the wavelength for reentry and promote AF initiation by unidirectional conduction block (Hansen et al., 2017), where studies in isolated atrial tissue from AF patients have confirmed substantial interstitial fibrosis to slow conduction (Krul S.P. et al., 2015). In computational studies, image-based models of fibrotic atria from AF patients with persistent AF demonstrate that regions with high fibrosis density and entropy perpetuate AF reentrant drivers (Zahid et al., 2016), where slowed conduction enables reentrant circuits in relatively small regions of the atria (Morgan et al., 2016). Thus, conduction slowing from ACh, in combination with impaired conduction from interstitial fibrosis, could further reduce the wavelength for reentry to increase AF susceptibility.

We hypothesize that RMP hyperpolarization by ACh delays atrial activation to facilitate AF in fibrotic atria with impaired conduction (Krul et al., 2014). To test this hypothesis, we performed computer simulations with models of a left atrial appendage (LAA) myocyte, a monolayer of LAA myocytes, and an intact left atrium (LA) to determine the mechanism by which ACh alters conduction and leads to AF. We then administered ACh to isolated human atrial myocytes and excised LAA from AF patients to corroborate the computer simulations.

## Materials and Methods

### Simulation Study

#### Computational Atrial Models

##### LAA myocyte

To simulate the electrophysiology of LAA myocytes in patients with persistent AF, the Courtemanche-Ramirez-Nattel model (Courtemanche et al., 1998) was modified according to the section “S1S2 Pacing and Arrhythmia Induction Protocol” in Bayer et al. (2016). Specifically, the maximal conductance for ion channels was modified to simulate electrical remodeling from AF (G_*to*_^∗^0.3, G_*Kur*_^∗^0.5, and G_*CaL*_^∗^0.3) and regional differences in LAA cellular electrophysiology (G_*Kr*_^∗^1.6, G_*Na*_^∗^2.0, and G_*K*__1_^∗^0.8). The dependence of transmembrane potential (V_*m*_) on ACh concentration was represented with the I_*KACh*_ formulation by Kneller et al. (2002). To simulate APD and RMP consistent with human atrial myocytes administered ACh (Table 1), I_*KACh*_ was modified according to “Supplementary Section 1: Acetylcholine-Activated Potassium Current” in Supplementary Material.

**TABLE 1:** Action potential characteristics during S1 pacing for LAA AF single-cell simulations and isolated human atrial myocytes from heart failure patients without AF.

**ACh (μM)**	**0.0**	**0.001**	**0.01**	**0.1**	***p*-value**
**Simulations: AF**					
APD (ms)	160	159	145	110	
V_*max*_ (mV/ms)	348	362	371	373	
RMP (mV)	−79	−83	−85	−86	
**Experiments: Non-AF**					
APD (ms)	370 ± 57			322 ± 74	0.191
V_*max*_ (mV/ms)	414 ± 51			420 ± 70	0.777
RMP (mV)	−73 ± 3			−77 ± 3	0.048

##### LAA monolayer

The 4 cm × 4 cm LAA monolayer model contained 14884 nodes and 29282 triangular finite elements with a fixed average edge length of 330 μm (same as LA model). A uniform left to right fiber direction was assigned to each LAA mesh element to account for anisotropic propagation of electrical waves in atrial tissue. Intracellular tissue conductivities for the directions parallel and perpendicular the cardiac fibers were adjusted so that CV matched that in the LAA of AF patients (Krul S.P. et al., 2015). See below and online ‘‘Supplementary Section 2: Conduction Velocity in the Left Atrial Appendage Tissue Model,” in Supplementary Material for details. Heterogeneous parasympathetic activation was included in the LAA model according to human LA studies (Vigmond et al., 2004; Chevalier et al., 2005). See “Supplementary Section 3: ACh Heterogeneity in the Left Atrial Appendage and Left Atrium Models,” in Supplementary Material for details.

##### LA

The LA from a bilayer model of 3D-imaged human atria (Labarthe et al., 2014) was used to determine if the effects of ACh on action potential dynamics and conduction led to sustained AF in the presence of interstitial fibrosis, altered tissue conductivity, and/or heterogeneous parasympathetic activation. This specific LA model was used because it simulates clinical AF with minimal computational load (Bayer et al., 2016). Regional differences in ion channel conductance were incorporated according to the section “S1S2 Pacing and Arrhythmia Induction Protocol” in Bayer et al. (2016), and heterogeneous parasympathetic activation was incorporated according to “Supplementary Section 3: ACh Heterogeneity in the Left Atrial Appendage and Left Atrium Models” in Supplementary Material. Image-based interstitial fibrosis from AF patients and tissue conductivities were assigned according to the following sections.

##### Tissue conductivity conduction settings

Tissue conductivity parameters for the LAA and LA models were adjusted so that monodomain simulation results matched conduction measurements in AF patients (Krul S.P. et al., 2015). Specifically, the longitudinal and transverse tissue conductivity parameters were adjusted so that longitudinal CV (CV_*L*_) was 44 cm/s and transverse CV (CV_*T*_) was 24 cm/s during pacing with a CL of 600 ms in both fibrotic and non-fibrotic LAA and LA models. Table 2 contains the conductivity values used for the LAA and LA simulations. Note, since the LAA and LA models share the same average mesh element edge length of 330 μM, the same conductivity values could be used for both. The default conductivities taken from Bayer et al. (2016) are labeled as non-AF in Table 2.

**TABLE 2:** Tissue conductivities in the LAA monolayer and LA models.

	***g*_l_ (S/m)**	***g*_t_ (S/m)**	**CV_L_ (cm/s)**	**CV_T_ (cm/s)**
**Fibrotic**				
AF	0.140	0.052	44.0	24.0
Non-AF	0.400	0.107	75.7	37.2
**Non-fibrotic**				
AF	0.107	0.045	44.0	24.0
Non-AF	0.400	0.107	96.8	45.1

*g_*l*_, longitudinal conductivity; g_*t*_, transverse conductivity; CV_*L*_, longitudinal conduction velocity; CV_*T*_, transverse conduction velocity. Conductivity values were determined with ACh = 0 μM. CV was computed using activation times calculated at steady-state during baseline pacing with a cycle length of 600 ms. Note, CV_*L*_ and CV_*T*_ for AF were taken from Krul S.P. et al. (2015).*

##### Interstitial fibrosis in the LAA and LA models

**FIGURE 1:**
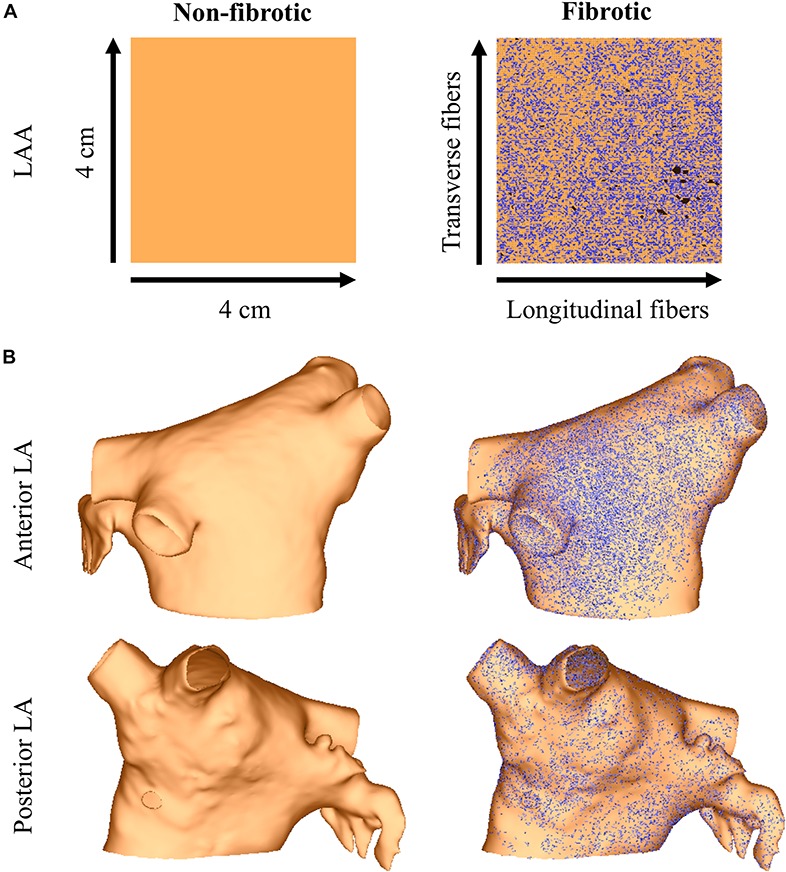
The LAA (panel **A**) and LA (panel **B**) models with and without image-based interstitial fibrosis. Active myocardium is in orange, inactive myocardium from interstitial fibrosis is in black (removed elements), and split element edges are in blue.

Image-based interstitial fibrosis from AF patients was incorporated into the LAA and LA meshes based on late-gadolinium enhancement imaging of persistent AF patients (Cochet et al., 2015). These intensity data were expressed as standard deviations above the mean of normal tissue (Oakes et al., 2009), and data from a 4 cm × 4 cm region of the LA were projected onto the LAA tissue model. Interstitial fibrosis, as described in the LAA of patients (mean age 58 years) with persistent AF (Krul S.P. et al., 2015), was then modeled as microstructural discontinuities that act as an interstitial conductive barrier (Bayer et al., 2016; Roney et al., 2016). Note, since myofibroblasts were not detected in the LAA samples with immunohistochemistry (Krul S.P. et al., 2015), (myo)fibroblast coupling was not included in the model. Briefly, mesh element edges were stochastically selected as fibrotic with a probability depending on the normalized late-gadolinium intensity value, and the direction of the mesh element edge was compared to the local fiber direction so that edges paralleling the longitudinal fiber direction (interstitial space) were four times more likely to be selected than edges transverse this direction. Selected mesh element edges were arranged into networks of connected edges and renumbered following a finite element approach (Costa et al., 2014). For each network, the renumbering that resulted in the fewest isolated mesh elements was used. Mesh elements for which all edges were selected, as well as unused nodes, were removed from the mesh. The final mesh was then assigned no flux boundary conditions along the strands of interstitial fibrosis. Interstitial fibrosis in the LAA and LA models using this approach is shown in Figure 1.

#### S1S2 Pacing and Arrhythmia Induction Protocol

S1S2 pacing was applied to each model at ACh concentrations of 0, 0.001, 0.01, and 0.1 μM. The maximum ACh of this range was chosen to reflect observations in human atrial myocytes (Table 1). For each concentration, the models were preconditioned by pacing the single-cell, center of the LAA monolayer, or tip of the LAA in the LA at a S1 cycle length (CL) of 600 ms for 100 beats using 2-ms-long stimuli at twice capture threshold. The models were then administered premature stimuli beginning with S2 CL = 400 ms, and then shortened by 10 ms decrements until loss of stimulus capture at the effective refractory period. For the minimum S2 CL with capture, the LAA and LA models were paced for 10 beats to test for unidirectional conduction block and reentry.

#### Action Potential and Conduction Parameters in the Single-Cell and Monolayer Models

For the last S1 and each S2 beat of the simulation pacing protocol, ATs were recorded when maximal dV_*m*_/dt occurred during the action potential upstroke. APD was recorded as the difference between AT and 90% repolarization (Bayer et al., 2016). The RMP was computed as the minimum V_*m*_ during the diastolic interval between action potentials. The maximum upstroke velocity (V_*max*_) was computed as the maximum + dV_*m*_/dt during the action potential upstroke (RMP to peak of the action potential). Total AT was the difference between the first and last activation in the LAA monolayer model.

#### Pseudo-ECG and Cycle Length of Reentry

Two reference points were chosen 3 cm away from the LA model center point. During S2 pacing and for 10 s after pacing, pseudo-ECGs were computed by taking the difference in extracellular voltage between these two points according to Gima and Rudy (2002). To compute the average CL of reentry for an episode of sustained AF, we detected all instances in time when the upstroke of the pseudo-ECG had dV/dt > 0.2 mV/ms, and then averaged the difference between all subsequently detected times.

#### Simulation Platform

Simulations were performed using the Cardiac Arrhythmia Research Package (Vigmond et al., 2008) on a single CPU of a generic desktop computer for single-cell simulations, and in parallel on two dual Hexa-Core Intel Xeon X5676 CPUs @3.06 GHz with 48 TB of memory for monolayer and LA monodomain simulations. To accurately compute V_*max*_, a time step and temporal output of 20 μs was used for single-cell simulations. All other simulations used a time step of 20 μs and a temporal output of 1.0 ms.

### Experimental Study

#### Human Atrial Myocyte Preparation

Human atrial myocytes were isolated from explanted hearts of 2 patients with end-stage heart failure at the time of cardiac transplantation. Informed consent was obtained and the protocol complied with institutional guidelines and the “Declaration of Helsinki.” Single myocytes from right (patient 1) and left (patient 2) atria were enzymatically isolated using a modified dissociation procedure (Amos et al., 1996). Right and left atria were collected in normal Tyrode’s solution containing (in mM) NaCl 140, KCl 5.4, CaCl_2_ 1.8, MgCl_2_ 1.0, glucose 5.5, and HEPES 5.0, at pH 7.4 (NaOH). The atrial tissue was cut into small strips (∼0.3 mm width, 0.5 mm length) and placed in a test tube containing nominally Ca^2+^-free Tyrode’s solution (20°C), which was refreshed two times. Subsequently, the strips were incubated for 20 min in nominally Ca^2+^-free Tyrode’s solution containing 1 mg/ml protease (220 U/l type XIV; Sigma, St. Louis, MO, United States) and 1 mg/ml BSA (Behring, Marburg, Germany). Next, the strips were placed for 20 min in nominally Ca^2+^-free Tyrode’s solution containing 1 mM EGTA, followed by an additional 45–60 min in nominally Ca^2+^-free Tyrode’s solution containing 0.2 mg/ml protease, 0.5 mg/ml collagenase (59 U/ltype B; Boehringer Mannheim, Mannheim, Germany), 0.5 mg/ml BSA, and 0.2 mM EGTA. The dissociation was stopped by transferring the strips into a modified Kraft-Brühe (KB) solution containing (in mM) KCl 85, K_2_HPO_4_ 30, MgSO_4_ 5.0, glucose 20, pyruvic acid 5.0, creatine 5.0, taurine 30, EGTA 0.5, ß-hydroxybutyric acid 5.0, succinic acid 5.0, Na_2_ATP 2.0, and polyvinylpyrrolidone 50 g/L at pH 6.9 (KOH). Single myocytes were obtained by gentle trituration through a pipette with a tip diameter of 1.0 mm for 5–10 min. The temperature of the dissociation solution was kept at 37°C. The KB solution containing single myocytes was placed in a disposable centrifuge tube in which the single myocytes were allowed to sediment. Finally, the KB solution was replaced by normal Tyrode’s solution in three steps. In each step, approximately 75% of the solution in the centrifuge tube was replaced by normal Tyrode’s solution (20–22°C). The interval between these steps was 10–15 min.

#### Data Acquisition and Analysis

Action potentials were recorded before and after administration of ACh (0.1 μM) at 36 ± 0.2°C in the ruptured whole-cell configuration of the patch-clamp technique using an Axopatch 200B amplifier (Molecular Devices Corporation, Sunnyvale, CA, United States). Signals were low-pass filtered with a cut-off frequency of 10 kHz and digitized at 25 kHz. Data acquisition and analysis were performed using custom software. Potentials were corrected for the liquid junction potential.

Action potentials were elicited at 1-Hz by 3-ms, 1.5–2 × threshold current pulses through the patch pipette. The bath solution contained Tyrode’s solution. The pipette solution contained (in mM) K-gluc 125, KCl 20, NaCl 5, and HEPES 10 at pH 7.2 (KOH). As in the single-cell simulations, we analyzed RMP, APD at 90% repolarization, and V_*max*_. Values from 10 consecutive action potentials were averaged.

### Clinical Study

#### Thoracoscopic Surgery

Thirteen LAAs were excised from AF patients during thorascoscopic, as described before (Krul et al., 2011), and immersed in cooled modified Tyrode’s solution before optical mapping. This study was in accordance with the declaration of Helsinki and approved by the institutional review board. All patients gave written informed consent.

#### Optical Mapping

Left atrial appendages were pinned down, stimulated near the base without epicardial fat (600 ms interval), and equilibrated for 30 min in a tissue bath perfused by oxygenated Tyrode’s solution (in mM) Na^+^ 155, K^+^ 4.7, Ca^2+^ 1.45, Mg^2+^ 0.6, Cl^–^ 136.6, HC0_3_^–^ 27, P$o_{4}^{3}$^–^ 0.4, glucose 11.1, and heparin 1000 IE at 36.5–37.5°C and pH = 7.4 (Krul S.P. et al., 2015). Then, the superperfused preparations were loaded with 4.4 μM Di-4-ANEPPS (Tebu Bio, Le Perray-en-Yvelines, France) and optical action potentials were recorded at 2 kHz using a MiCAM Ultima camera (SciMedia USA Ltd., Costa Mesa, CA, United States, 100 × 100 pixels). To reduce motion artifacts, 2–10 mM 2-3-butanedione monoxime (DAM, Sigma-Aldrich, B0753) was used, which was found to be more effective at eliminating motion artifacts in the LAA tissue preparations than Blebbistatin. Optical action potentials were recorded before and after 10 min of superfusion with 100 μM ACh (Lin et al., 2007) (A2261, Sigma) from seven preparations. The other six preparations without ACh served as the placebo to check for the rundown and time dependence of the model. The 500 ml of perfusate with ACh was recycled during the <15 min experiments with limited spontaneous breakdown (Sletten et al., 2005). Customized MATLAB (The MathWorks, Inc., Natick, MA, United States) software was used to determine local activations by selecting the steepest upstrokes of the optical action potential (Potse et al., 2002). Epicardial activation maps were constructed and CV calculated as described previously (Doshi et al., 2015).

#### Statistics

Data are presented as mean ± standard error of the mean unless stated otherwise, and categorical variables as percentages. An independent Student’s *t*-test was used to determine differences in normally distributed data, and the Pearson test for correlation. *p*-values (*p*) < 0.05 were considered statistically significant.

## Results

### ACh Alters Action Potential Dynamics

During S1 pacing, action potential dynamics in the LAA myocyte model changed gradually with respect to incremental ACh concentrations (Table 1). From 0 to 0.1 μM ACh, APD shortened by 41 ms, RMP hyperpolarized by 7 mV, and V_max_ increased by 27 mV/ms. In four isolated atrial cardiomyocytes from two patients with end stage heart failure (Table 1), ACh administration significantly lowered RMP from −73.3 ± 2.7 mV to −76.0 ± 3.2 mV (*p* = 0.048), which is an average hyperpolarization of 2.6 mV [95% confidence interval (CI): 0.05 to 5.20 mV]. APD was also shorter after ACh administration (370 ± 57 ms to 322 ± 74 ms), but this difference did not reach statistical significance (*p* = 0.191).

### ACh Delays Total Activation Time

**FIGURE 2:**
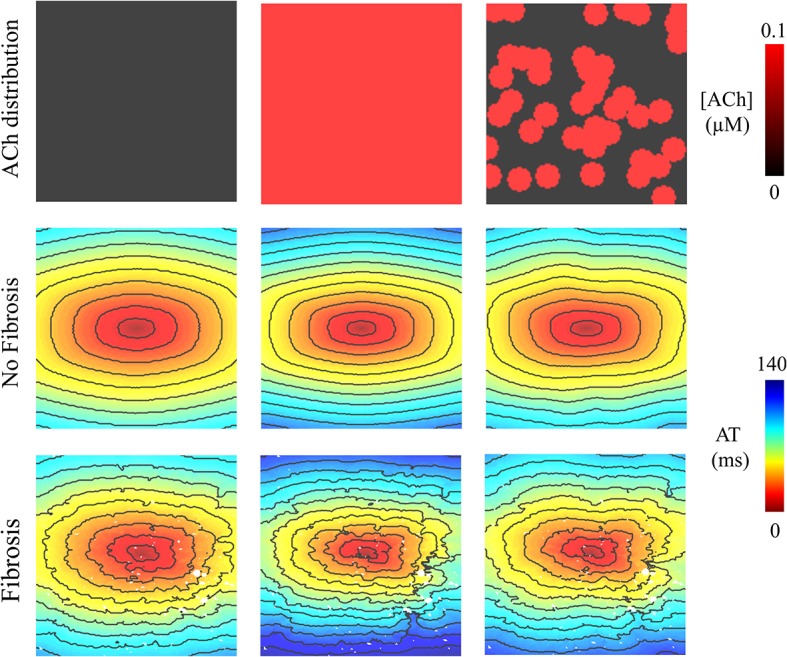
Activation maps in the 4 cm × 4 cm LAA monolayer without or with 0.1 μM homogenous/heterogeneous ACh (top row), and without or with fibrosis (middle and bottom rows). ATs are plotted for the last beat of S1 pacing. Isochronal lines are 10 ms apart. The AF conductivity values in Table 2 were used for these simulations.

In LAA monolayer models, 0.1 μM ACh delayed total AT during S1 pacing (compare left with middle and right columns of Figure 2). Without fibrosis (middle row of Figure 2), total AT prolonged from 90 ms to 112 ms for homogenous ACh, and to 103 ms for heterogeneous ACh. With fibrosis (bottom row of Figure 2), total AT prolonged even more from 104 ms to 139 ms for homogeneous ACh, and to 120 ms for heterogeneous ACh.

### ACh Shortens the Wavelength for Reentry

In LAA monolayer models, 0.1 μM ACh shortened λ by shortening APD and slowing conduction. Table 3 lists CV, APD, and λ along the longitudinal and transverse fiber directions of the LAA monolayer model for the last beat of S1 pacing (values inside parentheses), and for the minimum S2 CL with capture (values outside parentheses). The top of Table 3 refers to AF and the bottom refers to non-AF tissue conductivities in Table 2.

**TABLE 3:** Restitution properties for LAA slab simulations.

	**ERP ms**	**CV_L_ cm/s**	**CV_T_ cm/s**	**APD_L_ ms**	**APD_T_ ms**	**λ_L_ cm**	**λ_T_ cm**
**AF**							
F	230	28 (44)	16 (24)	161 (183)	160 (183)	4.46 (7.73)	2.60 (4.14)
F + HOMO	170	31 (33)	16 (17)	121 (135)	117 (130)	3.69 (4.37)	1.88 (2.19)
F + HET	180	28 (36)	16 (19)	135 (155)	140 (166)	3.76 (5.46)	2.23 (3.08)
NF	230	31 (44)	18 (24)	157 (182)	158 (182)	4.92 (8.15)	2.89 (4.53)
NF + HOMO	160	35 (38)	18 (19)	107 (120)	107 (119)	3.75 (4.49)	1.95 (2.30)
NF + HET	170	28 (39)	16 (21)	123 (148)	128 (162)	3.43 (5.79)	2.03 (3.36)
**Non-AF**							
F	230	47 (76)	26 (38)	157 (181)	157 (182)	7.35 (13.67)	4.14 (6.75)
F + HOMO	170	58 (64)	29 (31)	117 (132)	115 (130)	6.81 (8.36)	3.27 (3.98)
F + HET	180	46 (67)	25 (33)	133 (157)	139 (167)	6.07 (10.42)	3.44 (5.39)
NF	220	54 (97)	29 (45)	153 (180)	155 (181)	8.16 (17.42)	4.47 (8.15)
NF + HOMO	150	78 (88)	35 (39)	106 (120)	106 (119)	8.30 (10.50)	3.74 (4.59)
NF + HET	170	66 (90)	30 (40)	123 (150)	129 (163)	8.04 (13.42)	3.86 (6.54)

*CV_*L*_, longitudinal conduction velocity; CV_*T*_, transverse conduction velocity; APD_*L*_, longitudinal APD at 90% repolarization; APD_*T*_, transverse APD at 90% repolarization; λ_*L*_, longitudinal wavelength; λ_*T*_, transverse wavelength; F, fibrosis; NF, no fibrosis; HOMO, homogenous 0.1 μM ACh; HET, heterogeneous 0.1 μM ACh. Values listed inside the parentheses are for S1 pacing with a CL = 600 ms, and those outside parentheses for premature S2 with a CL just longer than ERP of the restitution pacing protocol. AF and non-AF: simulations with the AF and non-AF tissue conductivities in Table 2.*

In the fibrotic AF model with 0.1 μM homogeneous ACh (F + HOMO), the CV, APD, and λ were decreased compared to no ACh (F). For S1 pacing, the CV_*L*_ and APD_*L*_ decreased by 26%, and the CV_*T*_ and APD_*T*_ decreased by 29%. These reductions in both APD and CV shortened λ_*L*_ by 44%, and λ_*T*_ by 47%. For pacing with the minimum S2 CL, ACh shortened both λ_*L*_ and λ_*T*_ to below the LAA slab dimensions of 4 cm × 4 cm.

In the non-fibrotic AF model with 0.1 μM homogeneous ACh (NF + HOMO), the CV, APD, and λ were decreased when compared to the case without ACh (NF), and with similar magnitude to that in the fibrotic AF model. For S1 pacing, the addition of ACh decreased CV_*L*_ by 14%, APD_*L*_ by 34%, CV_*T*_ by 21%, and APD_*T*_ by 35%. These reductions in both APD and CV shortened λ_*L*_ by 45% and λ_*T*_ by 49%. For pacing with the minimum S2 CL, ACh shortened both λ_*L*_ and λ_*T*_ below the LAA slab dimensions.

**FIGURE 3:**
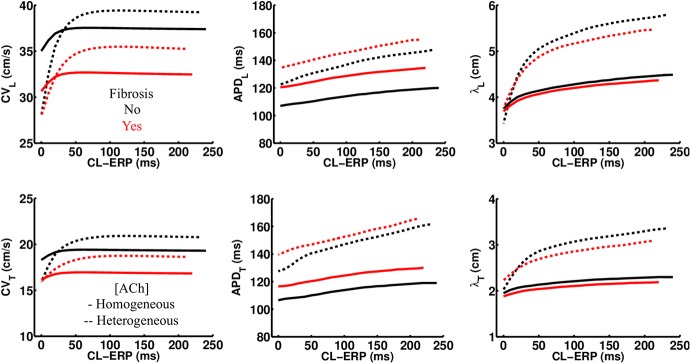
Restitution properties in the LAA tissue model with and without fibrosis, and for homogeneous and heterogeneous 0.1 μM ACh. The AF conductivity values in Table 2 were used for these simulations. Conduction velocity (CV), action potential duration at 90% repolarization (APD), and the wavelength for reentry (λ), were calculated along the longitudinal (L), and transverse (T) fiber directions. These parameters were plotted with respect to the S2 cycle length (CL) minus the effective refractory period (ERP).

**FIGURE 4:**
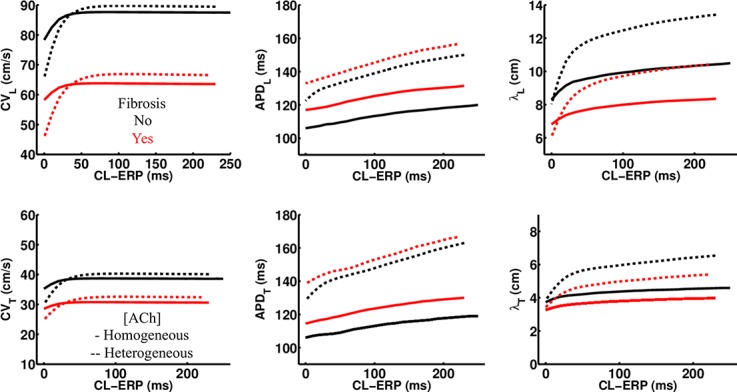
Restitution properties in the LAA tissue model with and without fibrosis, and for homogeneous and heterogeneous 0.1 μM ACh. The non-AF (faster conduction) conductivity values in Table 2 were used for these simulations. Conduction velocity (CV), action potential duration at 90% repolarization (APD), and the wavelength for reentry (λ), were calculated along the longitudinal (L) and transverse (T) fiber directions. These parameters were plotted with respect to the S2 cycle length (CL) minus the effective refractory period (ERP).

Regardless of whether ACh was administered homogeneously or heterogeneously, these decreases in CV, APD, and λ occurred in the LAA slab model both with and without fibrosis (compare NF + HOMO and F + HOMO with NF + HET and F + HET in the top of Table 3). For a visual comparison, the restitution curves for the AF conductivity values are shown in Figure 3, and the restitution curves for the non-AF conductivity values are shown in Figure 4.

### Heterogeneous ACh Leads to Unidirectional Conduction Block and Reentry

**FIGURE 5:**
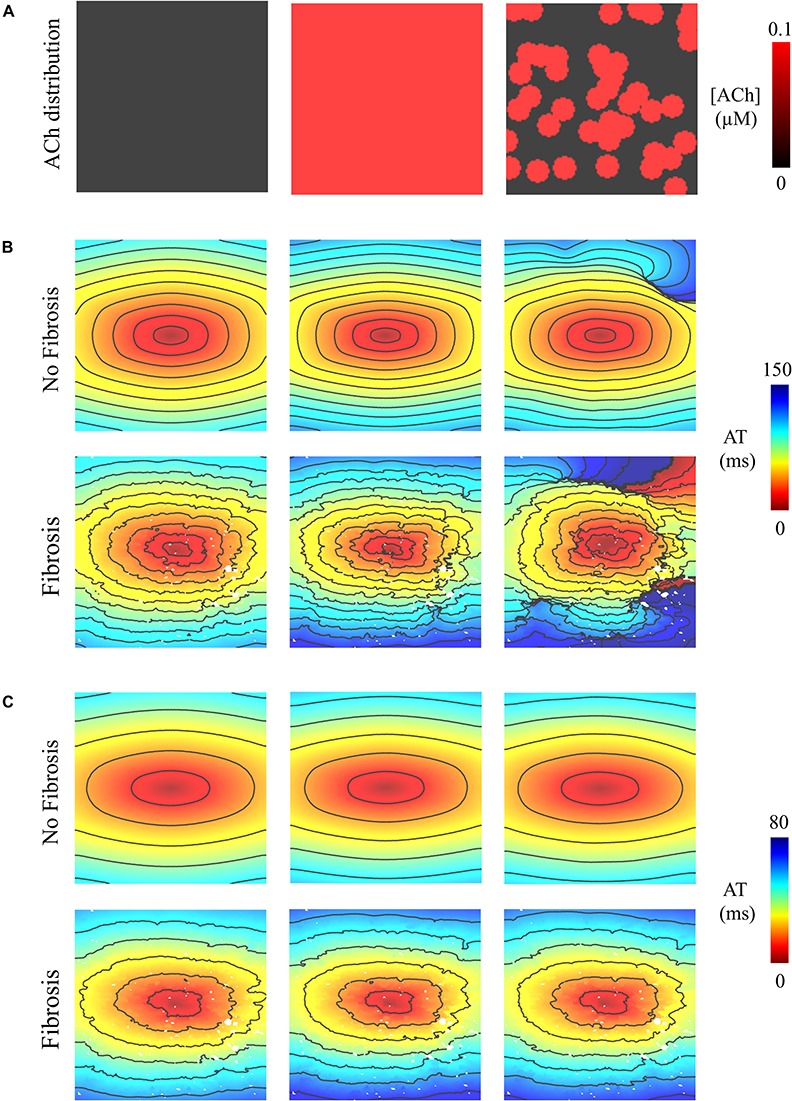
Activation maps in the 4 cm × 4 cm LAA model with and without fibrosis, and without, homogeneous, and heterogeneous ACh shown in **(A)**. ATs are plotted for the 10th beat during pacing with the minimum S2 CL. Isochronal lines are 10 ms apart. The AF conductivity values in Table 2 were used for the simulations in **(B)**, and the non-AF conductivity values (faster conduction) used for the simulations in **(C)**.

**FIGURE 6:**
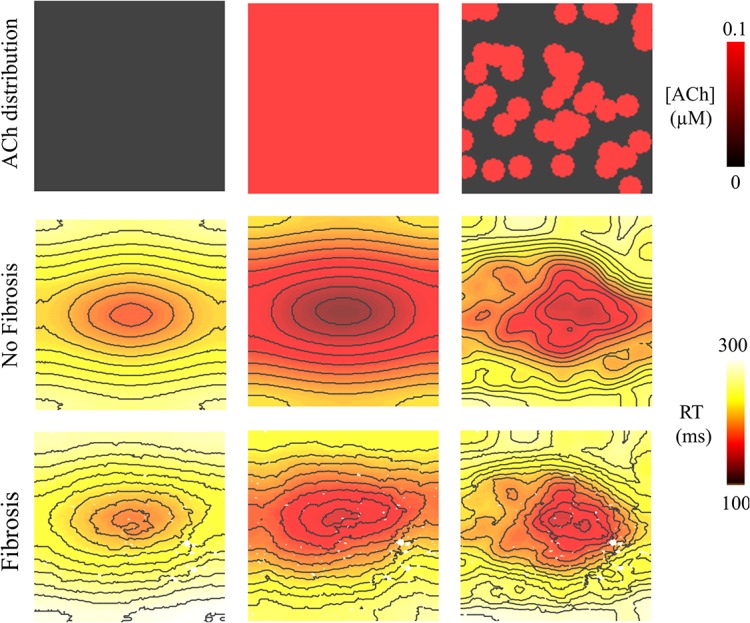
Repolarization time (RT = activation time + action potential duration) in the 4 cm × 4 cm LAA model with and without fibrosis, and with homogeneous and heterogeneous ACh. For ACh of 0.0 and 0.1 μM, activation times were plotted for the premature S2 with a CL above ERP or before conduction block and re-entry. Isolines are 10 ms apart. The AF conductivity values in Table 2 were used for these simulations.

Pacing at the minimum S2 CL resulted in unidirectional conduction block that was followed by reentry exclusively in the LAA monolayer model with 0.1 μM heterogeneous ACh. In Figure 5B, unidirectional conduction block occurred in the upper right-hand corners of the non-fibrotic and fibrotic LAA, which contained regions of tissue transitioning from 0.1 μM (clusters of red islands) to 0 μM ACh, which can be found in the far right panel of Figure 5A. The far right column of Figure 6 shows that repolarization time changed rapidly between these regions (crowded isolines next to sparse isolines), thereby generating steep gradients in repolarization. See “Supplementary Section 4: Simulation Movies,” in Supplementary Material for movies pertaining to the simulations in Figure 5B.

### Unidirectional Conduction Block and Reentry Is Inhibited by Faster Conduction

To determine whether delayed activation from heterogeneous ACh is essential for the unidirectional conduction block and/or reentry observed in Figure 5B, tissue conductivities in the LAA model were increased to the non-AF values shown in Table 2. Increasing conductivity in this manner increased CV in the LAA model by 150–220% (compare CV values in top versus bottom of Table 3). With faster conduction, conduction block no longer occurred with S2 pacing (Figure 5C). See “Supplementary Section 4: Simulation Movies” in Supplementary Material, for movies pertaining to the simulations in Figure 5C.

### Rapidly Pacing Fibrotic LA With Heterogeneous ACh Results in Sustained AF

**FIGURE 7:**
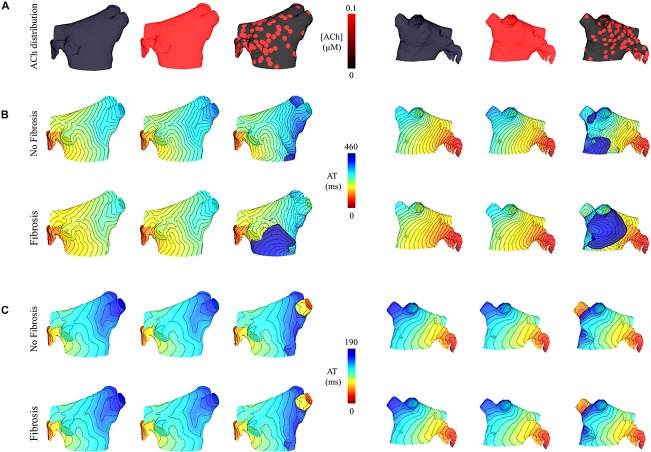
Posterior and anterior views of activation maps in the LA model with and without fibrosis, and without, homogeneous, and heterogeneous ACh shown in **(A)**. ATs are plotted for the 10th beat during pacing with the minimum S2 CL. Isochronal lines are 10 ms apart. The AF conductivity values in Table 2 were used for the simulations in **(B)**, and the non-AF conductivity values (faster conduction) used for the simulations in **(C)**.

**FIGURE 8:**
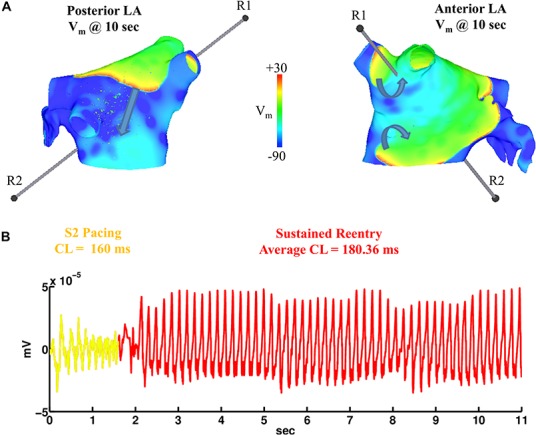
Figure-of-eight reentry on the anterior wall of the fibrotic LA model wit 0.1 μM heterogeneous ACh. The arrows in panel **(A)** indicate the directions of the propagating wavefronts. R1 and R2 are the reference electrodes used to compute the pseudo-ECG in panel **(B)**.

Simulations were repeated in the LA model under the same LAA monolayer model conditions to verify if ACh-induced reentry developed into sustained AF. At the middle and septal regions of the LA in Figure 7A, where the density of red islands with 0.1 μM ACh transitions from high to low, pacing at the minimum S2 CL led to unidirectional block and reentry (Figure 7B). However, sustained reentry that resembled AF in patients was only maintained in the LA containing both fibrosis and 0.1 μM heterogeneous ACh. Figure 8A shows a figure-of-eight reentry in the anterior LA that resembles AF in the pseudo-ECG after pacing at the minimum S2 CL (Figure 8B). The average CL of this reentry was 180 ms.

Figure 4C demonstrates that when using the faster conducting non-AF settings in Table 2, S2 pacing did not result in reentry from unidirectional conduction block. See “Supplementary Section 4: Simulation Movies” in Supplementary Material, for movies pertaining to the simulations in Figures 7B,C.

### ACh Delays Activation in LAA From AF Patients

**FIGURE 9:**
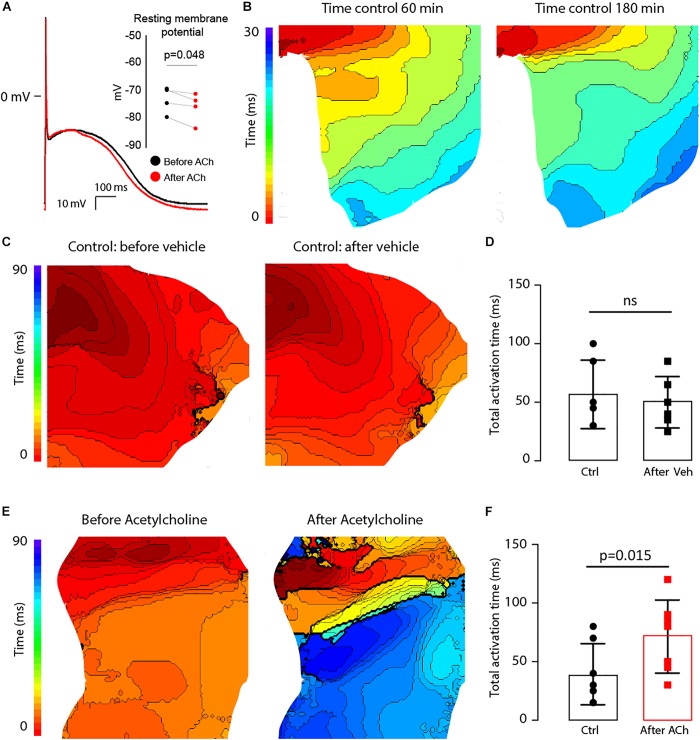
ACh at a concentration of 100 μM hyperpolarizes the resting membrane potential (RMP) and delays activation in the LAA of AF patients. **(A)** Typical action potential traces of an isolated atrial myocytes before (black) and after (red) ACh administration (*n* = 4). The bargraph (inset) shows the average RMP before and after ACh administration. **(B)** Epicardial activation patterns at 60 min and 180 min of superfusion indicating a stable condition of the tissue LAA preparation (*n* = 4). Isochronal lines are 2 ms apart **(C)**. Activation patterns of the LAA at the start of the experiment (right) and after 30 min (left). The bargraph in **(D)** shows that total activation time of the LAA preparation at the end of the experiment was not different from that at the beginning (*n* = 6). **(E)** Epicardial activation patterns before (right) and after incubation with ACh (left). Isochronal lines are 2 ms apart. The bargraphs in **(F)** shows that total activation time of the LAA preparation was prolonged after before and after ACh (*n* = 7).

The effect of lowering RMP and shortening APD (Figure 9) on atrial conduction was measured in isolated fibrotic LAA from AF patients (Table 4). To exclude possible ischemia as a result of superfusion of the LAA, instead of perfusion, optical action potentials were recorded for up to 180 min (Figure 9B). Total activation time (AT) of the preparation did not change over time (37.0 ± 11.0 vs. 40.0 ± 10.2, *n* = 4, *p* = 0.19), which suggests a normoxic state of the LAA. To further control for rundown of the LAA preparation, we then compared the effect of ACh (*n* = 6) on atrial conduction with the administration of a vehicle (*n* = 7), in this case H_2_O.

**TABLE 4:** Patient characteristics.

	**Patients (*n* = 13)**
Age, mean ± SD (range), years	60 ± 10 (43–78)
Male, n (%)	8 (62)
Years of AF, mean ± SD (range), years	5 ± 3 (1–11)
**AF type**	
Paroxysmal, n (%)	4 (31)
Persistent, n (%)	9 (69)
Previous PVI, n (%)	3 (23)
CHADSVASc, median, range	1 (0–7)
0–1	7 (54)
>2	6 (46)
**Left atrial size**	
Atrial volume index, mean ± SD (range), m^2^ml	40 ± 10 (26–58)
**Anti-arrhythmic medication**	
Flecainide, n (%)	7 (54)
Beta-blocker, n (%)	10 (77)
Sotalol, n (%)	2 (15)
Amiodarone, n (%)	1 (8)
Verapamil, n (%)	0 (0)

*PVI, pulmonary vein isolation; SD, standard deviation.*

Acetylcholine administration did not significantly reduce CV in the LAA preparations (61.6 ± 11.0 vs. 60.1 ± 8.3 cm/s, *n* = 6, *p* = 0.7). Figures 9C,E show epicardial activation patterns before and after administration of the vehicle or ACh. In the LAA preparations not administered ACh (vehicle), activation patterns did not differ between the two S1 pacing experiments, where the total AT went unchanged from 56.7 ± 29.3 ms to 50.0 ± 21.9 ms (*p* = 0.15, Figure 9D). However, activation patterns were different after ACh administration (Figure 9E) with total AT significantly prolonging from 39.3 ± 26 ms to 71.4 ± 31.2 ms (*p* = 0.036, Figure 9F), which is an average increase of 32 ms (95% CI: 3 to 61 ms). Due to the small dimensions of the LAA preparations (∼2 cm × ∼2 cm), we were unable to induce sustained reentry during the pacing protocol.

## Discussion

This study demonstrates that ACh delays activation in human atria to facilitate AF. In computer simulations, ACh delays activation by hyperpolarizing the RMP of atrial myocytes, which is enhanced by fibrosis and leads to AF in rapidly paced fibrotic LA with heterogeneous parasympathetic activation. Corroborating the simulations, ACh shortens APD in addition to hyperpolarizing RMP in human atrial myocytes, which delays atrial activation in the LAA of AF patients. These results support the hypothesis that patients with severe fibrosis and elevated vagal tone are more vulnerable to AF.

### ACh Delays Activation in Human Atria

In contrast to canine studies showing little effect of ACh on atrial conduction (Schuessler et al., 1991), similar ACh concentrations significantly delayed atrial activation in our computer models and patients. This agrees with previous human studies (Oliveira et al., 2011). Interestingly, we too did not directly observe CV slowing in the LAA of AF patients (8), even when obvious changes in activation were present. This discrepancy arises when measuring CV between two fixed points, since different activation patterns can generate the same CV, i.e., alternating fast/slow propagation can produce the same CV as uniform propagation. Thus, even though CV was unchanged by ACh in AF patients, total AT was prolonged from ACh, and likely more so at small isthmuses created by interstitial fibrosis present in the LAAs we measured (Krul S.P. et al., 2015).

### Mechanism of Delayed Atrial Activation

Over the past few decades, animal studies have reported ACh to shift RMP to more negative potentials in atrial myocytes (Pott, 1979; Molina et al., 2007; Verkerk et al., 2012). However, its effect on atrial conduction and arrhythmogenesis in humans is not fully understood. In this study, we demonstrate that a modest > −3 mV shift of RMP in human atrial myocytes delays activation by >30 ms in atrial tissue.

The shift of RMP by ACh to more negative potentials in our atrial models increased the potential difference between the RMP and threshold potential, which was apparent by the need to the increase stimulus current for S1 pacing by a factor of 1.4 in single cells and 1.3 in LAA and LA simulations after ACh administration. This larger potential difference reduces tissue excitability, which in turn could delay atrial activation (Dhamoon and Jalife, 2005), especially for patients with various degrees of fibrosis. Alternatively, delayed activation in the LAA of AF patients could have resulted from ischemia, though this was unlikely since we made sure the thickness of the LAA preparations did not exceed 800 μm, which is twice the diffusion limit of oxygen (Kang et al., 2016).

Another possible explanation for lowering RMP, one this study did not formally address, is the role of (myo)fibroblast coupling with atrial myocytes. Myofibroblasts, a differentiated form of the fibroblast, have been shown to connect to cardiomyocytes *in vitro* cell layer models (Miragoli et al., 2006; Rohr, 2012). Past *in silico* studies suggest (myo)fibroblasts to act as a passive electric load that depolarizes cardiomyocyte RMP through gap junctional coupling with myocytes (Xie et al., 2009). Therefore, if ACh could inhibit this coupling, RMP would decease. The effects of (myo)fibroblast coupling in response to ACh warrants further investigation.

### Mechanism of ACh Induced AF

Despite minimal changes to global conduction velocity, heterogeneous parasympathetic activation significantly alters local conduction. In human atria, parasympathetic activation is heterogeneous (Chevalier et al., 2005), which generates steep repolarization gradients that locally slow conduction enough to promote reentry via unidirectional conduction block of wavefronts traveling from regions with ACh to regions without ACh (Figure 6). Under certain circumstances, this could make the atria vulnerable to reentrant arrhythmia from high-frequency ectopic foci (Arora, 2012). In computer models and AF patients, we show that ACh can delay atrial activation. When heterogeneous ACh delays activation and shortens APD, this combination increases the dispersion of repolarization, which would contribute to the onset of AF in patients (Vigmond et al., 2004).

In computer models, ACh heterogeneity led to the unidirectional conduction block of wavefronts traveling from regions of high to low ACh (Figures 5B, 7B). At these regions, there is a steep gradient in repolarization time (Figure 6), which is arrhythmogenic during high-frequency pacing (Bayer et al., 2016), and is consistent with computational studies that vary ACh concentration in a time-dependent manner (Matene et al., 2014). In combination with further conduction slowing from interstitial atrial fibrosis, unidirectional conduction block at regions with ACh heterogeneity led to sustained reentry that resembled AF (Figure 8), which had an average CL within the clinical range observed for patients with persistent AF (Haissaguerre et al., 2014).

In patients, advanced atrial fibrosis coincides with persistent AF as shown in Table 4 of Dzeshka et al. (2015). Although atrial fibrosis may explain the maintenance and complexity of AF, it does not fully explain the initiation of AF. Our study shows that heterogeneous conduction slowing from ACh, in combination with impaired conduction from atrial fibrosis, is a plausible arrhythmogenic substrate underlying the onset of AF in patients with elevated vagal tone.

### Clinical Implications

There are three potential clinical implications from these findings. First, preventing delayed activation from the presence of ACh may be a promising therapeutic approach to decrease the probability of parasympathetic mediated AF. This could be achieved by modulating RMP directly via I_*KACh*_, the inward rectifier potassium current I_*K*__1_, and/or the sodium-calcium exchanger NCX. Alternatively, conduction could be improved by desensitizing atrial myocytes to ACh, which could be achieved by mutating atrial specific muscarinic receptors using CRISPR-Cas9, or by enhancing cellular coupling to increase the electrical conductivity of the tissue, which could be effected by upregulating the expression of atrial specific connexins. In any case, future work is warranted to explore the development of such precision engineered therapies for patients susceptible to AF.

Second, if the effects of atrial fibrosis on conduction are irreversible, AF treatments could suppress the parasympathetic nervous system. Several studies have shown the suppression of the parasympathetic nervous system in the posterior LA (Arora et al., 2008), and/or the ligament of Marshall (Ulphani et al., 2007), to prevent AF. This may be feasible with gene therapy applied to the left atrium via constitutive expression of Gα_*i/o*_ by non-viral plasmid vectors (Aistrup et al., 2011).

Third, clinical interventions using radiofrequency ablation to mediate the effects of the parasympathetic nervous system may potentially form an anti-arrhythmic strategy for the treatment of parasympathetically mediated AF (Krul S.P.J. et al., 2015). For example, endocardial ablation of the ganglionic plexuses could decrease parasympathetic effects on the sinoatrial and atrioventricular nodes. However, this would result in a large endocardial scar. Thus, it is unclear whether the lower rate of AF recurrence would be due to a more thorough atrial ablation or parasympathetic modulation.

### Comparison With Previous Computational Studies

The computational models used for this study differ from our previous studies in a few ways. First, APD restitution is relatively flat at short pacing CLs (Figures 3, 4) when compared to patients administered Adenosine (Krummen et al., 2012). This is due to the fact that baseline APD is shorter in the models for this study (119–183 ms) than in those in the previous study (∼200 LA body). This is the result of using an ACh concentration that reduces ADP by >50 ms (Table 1 and Figure 9A), rather than by only 20 ms in the study using Adenosine to elevate ACh concentrations.

Second, baseline longitudinal CV was 62 cm/sec in our previous thin 3D cable model, which represents conduction in normal tissue without fibrosis. Based on recent evidence showing significant fibrosis in the LAA tissue models used for this study that slows conduction (Krul S.P. et al., 2015), we slowed CV_*L*_ and CV_*T*_ via altering tissue conductivities and finite element mesh edge-splitting to reflect conduction slowing from fibrosis. To be sure, we still tested the faster ranges of CV reported elsewhere under different conditions by running simulations (Table 3) with the values listed in the bottom portion of Table 2.

Lastly, we used a modified formulation for I_*KACh*_. To adapt the I_*KACh*_ formulation to human atrial myocytes, we performed single-cell experiments with 0.1 μM ACh to observe changes in APD and RMP (Figure 9A and Table 1). In these experiments, APD shortened on average by 50 ms and RMP was more negative by 4 mV when administered 0.1 μM ACh. In the I_*KACh*_ formulation by Kneller et al. (2002), which was fit to data from right atrial canine myocytes, Figure 2A in their publication shows that 0.1 μM ACh shortens APD by >200 ms. To account for this discrepancy, we decreased the sensitivity of I_*KACh*_ to ACh in order to shorten APD according to the human atrial myocyte experiments. We then modified the voltage-dependence of the I_*KACh*_ I-V relationship to account for a more negative RMP in human atrial myocytes. To fully justify all changes to the I_*KACh*_ formula, future patch-clamp studies should be performed to validate this formulation in human atrial myocytes, and then perform microelectrode recordings in tissue to obtain dose-dependent responses for APD and RMP to ACh in order to compare with the single-cell studies.

### Limitations

In the computational study, spatial ACh heterogeneity is determined in a rule-based fashion using histological data from patient LA (Chevalier et al., 2005) (see “Supplementary Section 3: ACh Heterogeneity in the Left Atrial Appendage and Left Atrium Models” in Supplementary Material). In order to obtain a more accurate representation of spatial heterogeneity in the parasympathetic nervous system, novel 3D imaging modalities need to be utilized. Second, the spatial density of active muscarinic receptors is not known for the entire LA. Therefore, it was assumed that all regions of the models with elevated ACh responded equally. Future studies should stain for muscarinic receptors throughout the LA to investigate how spatial muscarinic receptor density correlates to repolarization heterogeneity.

In the clinical study, due to ethical reasons we could only obtain LAAs from patients who underwent surgery for AF. Thus, we were unable to perform studies on LAA and single-cells from healthy patients. Second, the antiarrhythmic drugs the patients received may have influenced the electrophysiological remodeling of the LAA preparations. Third, the dose-response for ACh concentrations in patients versus models is not necessary equivalent, though we did use a very high ACh concentration in the LAA of patients to compare with the maximal effects of ACh in the computer simulations. Future studies should perform microelectrode recordings to confirm these changes in action potential dynamics from ACh in intact human atrial tissue, as well as to investigate the changes in I_*KACh*_ before and after administering ACh to human atrial cells. Fourth, action potential upstroke velocities are slower in cardiac tissue optically imaged with voltage-sensitive dyes, which can also increase over time (Hyatt et al., 2003). This could potentially impact our activation (latency-to-fire) and conduction velocity (excitability) measurements in the LAA preparations. However, previous studies not using voltage-sensitive dyes (Boukens et al., 2013; Gillers et al., 2015) report similar conduction velocities (longitudinal CV of 55–60 cm/s) to those not using voltage-sensitive dyes (Remme et al., 2009; Bakker et al., 2012). Additionally, Figures 9B,C show stable ATs for up to 180 min. Fifth, mechanical uncouplers as 2-3-butanedione monoxime could have electrohysiological effects on our LAA tissue preparations (Watanabe et al., 2001). However, activation patterns in Figure 9C did not change over time, though in Figure 9E activation patterns changed markedly after ACh administration (panels in Figure 9E), indicating 2-3-butanedione monoxime had little effect on the activation patterns in our studies. Sixth, the 100 μM dose of ACh in the LAA preparations was chosen to reflect previous AF studies (Lin et al., 2007). It is possible high ACh concentrations could desensitize muscarinic receptors and render the LAA tissue non-excitable to promote conduction slowing and/or block. Lastly, relative changes in RMP and APD were based on isolated human atrial myocytes from heart failure patients without AF. Thus, relative changes in RMP and APD in response to ACh may differ between patients with and without AF, as well as, for patients with and without heart failure. Since baseline APD (Fedorov et al., 2011) and ERP (Sanders et al., 2003) in failing atria are longer when compared to non-failing and AF conditions, we only preserved the absolute decrease in APD before and after ACh between our experiments and simulations.

## Conclusion

Acetylcholine delays activation to facilitate AF in fibrotic atria with heterogeneous parasympathetic activation. Delayed activation results from a shift in RMP by ACh to more negative potentials, and is enhanced by fibrosis. Rapidly pacing fibrotic atria with heterogeneous parasympathetic activation leads to AF from unidirectional conduction block and sustained reentry at regions that transition from high and low ACh. The results of this study predict that AF patients with severe fibrosis are more vulnerable to parasympathetically mediated AF, and that these patients would likely benefit from clinical treatments that prevent delayed activation and/or suppress the parasympathetic nervous system.

## Ethics Statement

This study collected human data with a protocol previously approved by the AMC group (Krul et al., 2011). As stated in the manuscript, this study was performed in accordance with the Declaration of Helsinki and approved by the institutional review board. All patients gave written informed consent.

## Author Contributions

All authors have made substantial contributions to the conception and/or design of the work and have approved the final version to be published while agreeing to be accountable for all aspects of the work to ensure that questions related to the accuracy or integrity of any part of the work are appropriately investigated and resolved. JB, CR, and EV provided the simulation data. SK, AD, WB, NB, RB, and JG provided the patient data. AV provided the single-cell data. JB, SK, BB, RC, and JG performed an in depth analysis of all the data and drafted the manuscript. JB, SK, BB, CR, AD, WB, NB, AV, EV, RC, and JG revised the manuscript during the peer-review process.

## Conflict of Interest Statement

The authors declare that the research was conducted in the absence of any commercial or financial relationships that could be construed as a potential conflict of interest. The reviewer OA declared a shared affiliation, with no collaboration, with one of the authors, CR, to the handling Editor at the time of the review.
