# Supplementary material for: Acetylcholine Delays Atrial Activation to Facilitate Atrial Fibrillation
Source: Front Physiol. 2019 Sep 4;10:1105. doi: 10.3389/fphys.2019.01105 (PMC6737394; doi:10.3389/fphys.2019.01105)
Supplement: Supplementary file 1 [file Data_Sheet_1.PDF]

# SUPPLEMENTAL MATERIAL

# Abbreviations:

ACh – Acetylcholine

AF – Atrial Fibrillation

APD – Action Potential Duration

AT – Activation Time

CL – Cycle Length

CV – Conduction Velocity

$I_{KACh}$ – Acetylcholine-activated potassium current

mCRN - modified Courtemanche-Ramirez-Nattel

ERP – Effective Refractory Period

L – Longitudinal

LA – Left Atrium

LAA – Left Atrial Appendage

RMP – Resting Membrane Potential

T – Transverse

$V_m$ – Transmembrane Potential

$V_{max}$ - Maximum Upstroke Velocity

$\lambda$ – Wavelength for reentry

# Supplemental Section 1: Acetylcholine-activated potassium current

In atrial myocytes, acetylcholine (ACh) activates the outward potassium current ($I_{KACh}$) in a dose-dependent manner to shorten action potential duration (APD) (Farges, Ollagnier, and Faucon 1977) and to shift the resting membrane potential (RMP) to more negative potentials (Verkerk et al. 2012, Molina et al. 2007). To simulate these effects from ACh on the cellular electrophysiology of atrial myocytes in our computational studies, we used the mathematical formulation for $I_{KACh}$ from Kneller et al (Kneller et al. 2002) in the Courtemanche-Ramirez-Nattel (CRN) model (Courtemanche, Ramirez, and Nattel 1998) under persistent atrial fibrillation (AF) conditions in the human left atrial appendage (LAA) (Bayer et al. 2016). This required two modifications to $I_{KACh}$ with each described below.

First, in human atrial myocytes APD shortens by >50 ms when administered 0.1 μM [ACh], see Figure 9.A and Table 1 in the manuscript. The $I_{KACh}$ formulation by Kneller et al (Kneller et al. 2002) was fit to data from right atrial canine myocytes. However, in Figure 2.A of their manuscript, it shows that 0.1 μM [ACh] shortens APD by > 200 ms in their model, which was confirmed in our model as well (Figure S1.A). In order to shorten APD consistent with human atrial myocytes at [ACh] ≤ 0.1 μM, we decreased the sensitivity of $I_{KACh}$ to ACh by multiplying [ACh] in Equation 1 below by a factor of $10^{-1}$.

$$I_{KACh} = \left(\frac{10.0}{1+\frac{9.14}{([ACh]*\mathbf{10^{-1}})^{0.478}}}\right) * \left(0.05 + \frac{0.45}{1+\exp\frac{V_m+59.54}{17.18}}\right) * (V_m - E_K) \qquad \text{(eq. 1)}$$

In Equation 1, $V_m$ is the membrane potential and $E_K$ is the Nernst potential for potassium.

Secondly, human atrial myocytes exhibited on average a more negative RMP by 4mV when administered 0.1 μM [ACh], see Figure 9.A and Table 1 in the manuscript. This has also been observed in rabbit and trout atrial cells for ACh concentrations that shorten APD by > 30 ms (Verkerk et al. 2012, Molina et al. 2007). Since the sensitivity of $I_{KACh}$ to [ACh] was decreased, the $I_{KACh}$ formulation in Equation 1 underestimates this change in RMP at 0.1 μM [ACh] by ~50%. Specifically, only a -2 mV difference in RMP is observed in the original Kneller $I_{KACh}$ formulation when [ACh] shortens APD by ~50 ms (Figures S1.B and S1.C). Thus, the voltage dependent parameters of $I_{KACh}$ were increased until RMP was more negative by >4 mV at an [ACh] of 0.1 μM. The voltage-dependent parameters adjusted for $I_{KACh}$ in Equation 1 are highlighted in bold for Equation 2.

$$I_{KACh} = \left(\frac{10.0}{1+\frac{9.14}{([ACh]*10^{-1})^{0.478}}}\right) * \left(0.05 + \frac{5.0}{1+\exp\frac{Vm+85.0}{5.0}}\right) * (V_m - E_K) \qquad \text{(eq. 2)}$$

Figure S1 shows the dose-dependency of the original $I_{KACh}$ formulation by Kneller et al versus the modified formulation in Equation 2 for human atrial myocytes administered 0-0.1 μM [ACh] during pacing at a cycle length (CL) of 600 ms.

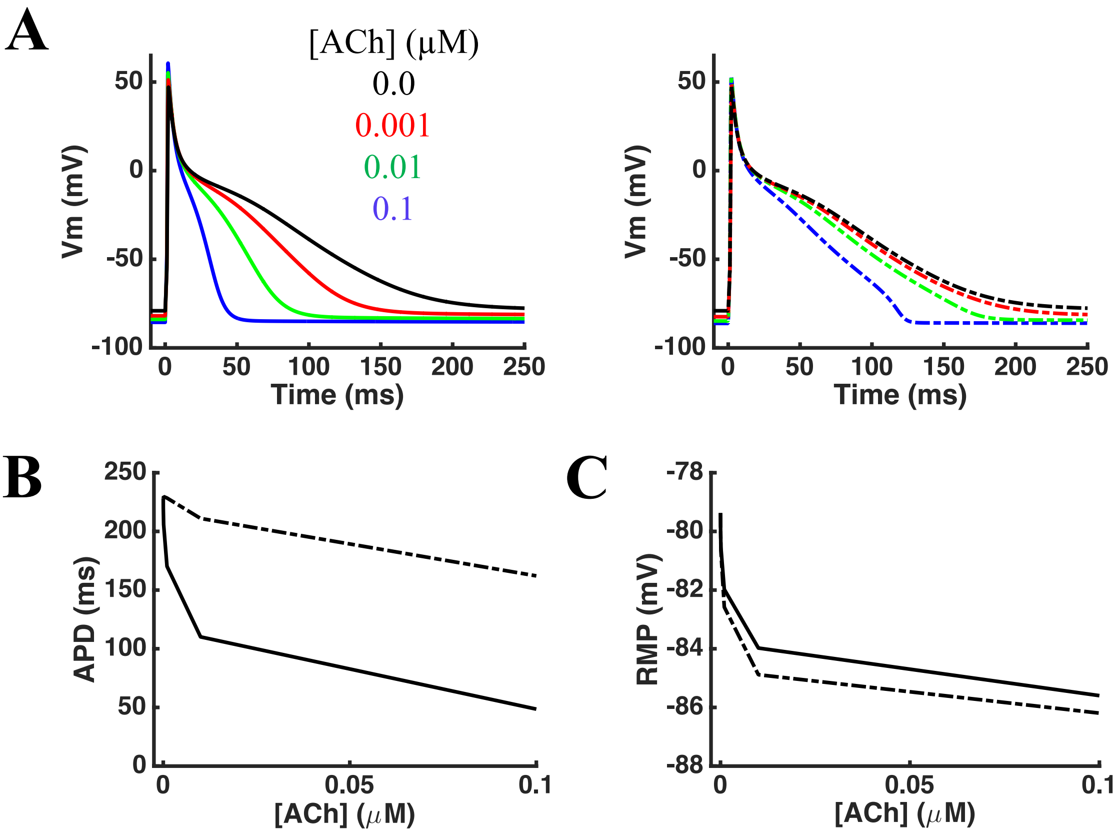


**Figure S1**: Dose-response to [ACh] for A.) membrane potential ($V_m$), B.) action potential duration (APD) and C.) resting membrane potential (RMP) at steady-state during pacing with a cycle length of 600 ms. Plots with solid lines used the original Kneller $I_{KACh}$ formulation, and plots with dashed lines used the $I_{KACh}$ formulation in Equation 2 modified for human atrial myocytes.

# Supplemental Section 2: Conduction velocity in the left atrial appendage tissue model

Average conduction velocity (CV) along the longitudinal ($CV_L$) and transverse ($CV_T$) fiber axes in the LAA slab model was computed as described below. At the same locations, APD was averaged for the longitudinal ($APD_L$) and transverse ($APD_T$) directions in the LAA model. The longitudinal and transverse wavelength of reentry ($\lambda$) were calculated as $\lambda_L = CV_L * APD_L$ and $\lambda_T = CV_T * APD_T$, respectively.

Activation times were computed at eight locations in the LAA tissue model at the moment in time when the change in the membrane potential ($dV_m/dt$) during the action potential upstroke was maximal. The first four locations were 0.5 cm from the center of the LAA tissue model along the longitudinal (L1’,L2’) and transverse (T1’,T2’) directions. The next four locations were 1.5 cm away from the center of the LAA tissue model along the longitudinal (L1,L2) and transverse (T1,T2) fiber directions. These locations are illustrated in Figure S2.

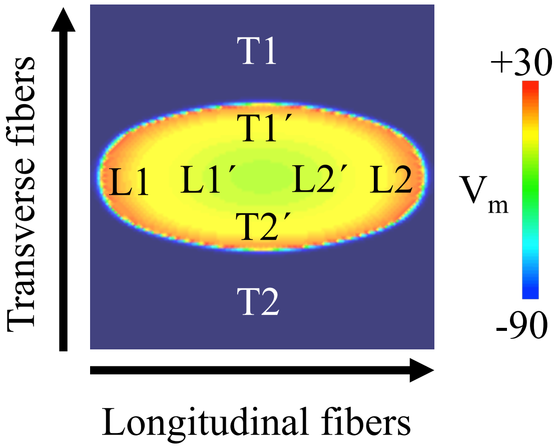


**Figure S2:** Electrical propagation initiated at the center of the LAA model. Activation times are determined at 0.5 cm and 1.5 cm away from the center of the LAA model at the eight labeled locations along the longitudinal (L1’,L2’,L1,L2) and transverse (T1’,T2’,T1,T2) fiber directions.

CV in the LAA model was computed using the activation times at the LAA locations shown in Figure S2 according to equations 3 and 4.

$$CV_L = \frac{(L1 - L1') + (L2 - L2')}{2D} \qquad \text{(eq. 3)}$$

$$CV_T = \frac{(T1 - T1') + (T2 - T2')}{2D} \qquad \text{(eq. 4)}$$

D is the 1cm distance between the paired points, and $CV_L$ and $CV_T$ represent averaged CV along each the longitudinal and transverse directions, respectively. The average CV was taken since conduction is not guaranteed to be uniform in the presence of fibrosis and/or ACh heterogeneity. Therefore, APD and $\lambda$ in the LAA were also averaged.

# Supplemental Section 3: ACh heterogeneity in the left atrial appendage and left atrium models

Parasympathetic activation in the LAA and LA is heterogeneous (Chevalier et al. 2005, Arora et al. 2007). Vagal induced dispersion of APD via heterogeneous ACh can be arrhythmogenic (Liu and Nattel 1997, Vigmond et al. 2004). Therefore, heterogeneous parasympathetic activation was included in the LAA and left atrium (LA) models by applying islands of [ACh] with 2.4 mm radii according to Vigmond et al (Vigmond et al. 2004), and a non-uniform spatial distribution as reported in human LA (Chevalier et al. 2005). As in Vigmond et al (Vigmond et al. 2004), we also tested radii of 1.6 and 3.2 mm. Conduction slowing and block occurred for only radii 2.4 and 3.2 mm, and the results between the two did not notably differ. So we only report results for the radius of 2.4 mm here and in the manuscript.

To implement this ACh heterogeneity, the solution to Laplace's equation was solved for in the LAA model with Dirichlet boundary conditions of 0 applied to its center, and 1 to its four outer corners. For the LA model, the Dirichlet boundary condition of 0 was applied at the tip of the LAA, and 1 along a line connecting the right inferior pulmonary vein with the fossa ovalis. The solutions to Laplace's equation with these Dirichlet boundary conditions are shown in Figure S3.A, and were solved for using the methods in Bayer et al (Bayer et al. 2012). Using these Laplace solutions, the LAA and LA models were then subdivided into three regions to apply the ACh heterogeneity derived from human LA (Figure S3.B). First, the width of each subdivision (cm) was computed from the center to the perimeter of the LAA model, and from the right to left of the LA model. Second, the number of ACh islands to assign to each subdivision was determined by multiplying the subdivision's width by the clinically derived densities of 28 islands/cm for the LAA's center and LA's left-side, 24 island/cm for the middle region of both models, and 13 island/cm for the outer region of the LAA and the LA's right-side. Lastly, each island was assigned to a mesh node chosen at random within the subdivisions at the densities above. The results of applying this approach to the LAA and LA models are shown in Figure S3.C.

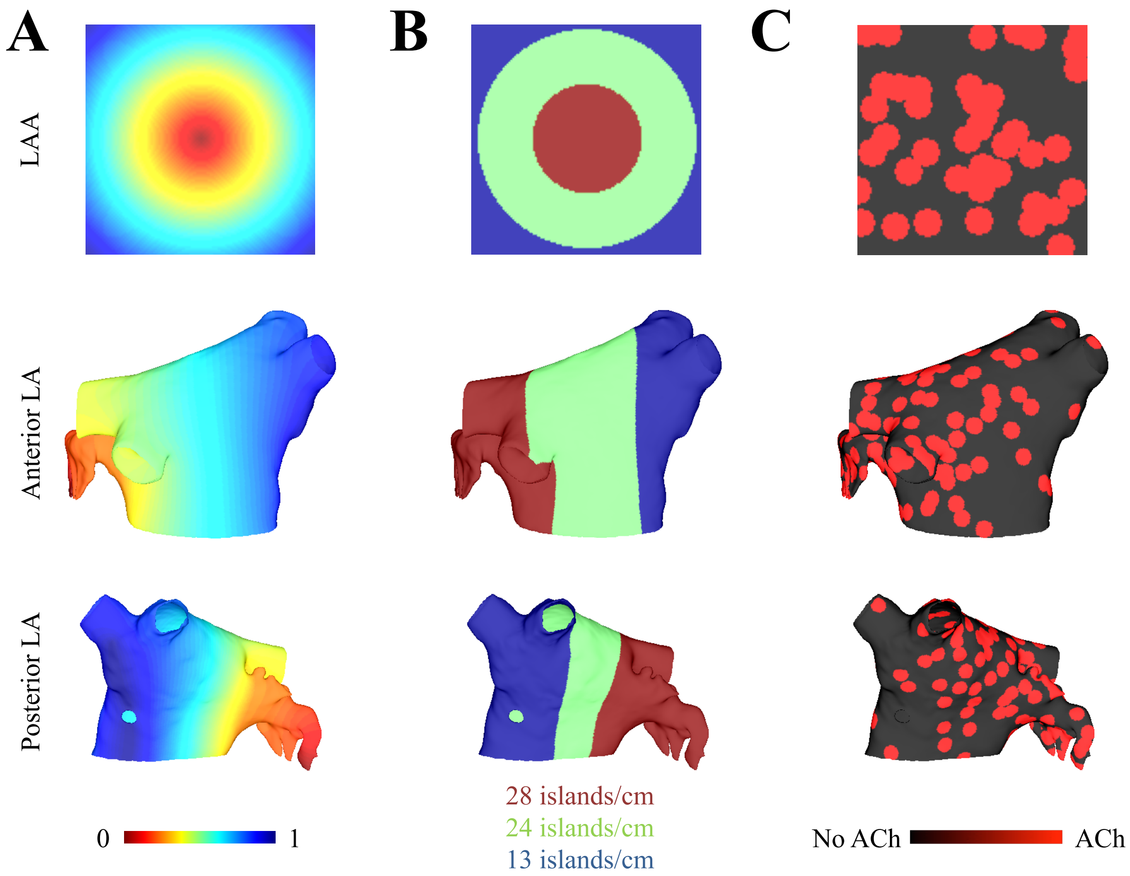


**Figure S3:** A.) Solutions to Laplace's equation with Dirichlet boundary conditions of 0 applied to the region in dark red, and 1 at the region of dark blue, of the LAA and LA models. B.) Subdivisions of the LAA and LA models to apply ACh islands according to clinical data(Chevalier et al. 2005), where the number of ACh innervations per 1 cm wide cross-sections taken from the center to the perimeter of the LAA model, and from the right- to left-sides of the LA. C.) Island distributions to administer [ACh]. Note, the red islands are denser at the center of the LAA and left-side of the LA models, and less dense on the perimeter of the LAA and right-side of the LA models.

# Supplemental 4: Simulation movies

Please download the supplemental movies from the journal's website. The movies will either have the filename "ACh_supplemental movies_SX.pptx" or "Presentation X.PPT", with X being the number 1-12 described below.

Movies S1-S6 correspond to the simulations in Figure 5 of the manuscript, which show changes in the membrane potential during pacing with the minimum S2 pacing CL. The left panel of these movies corresponds to simulations with the AF conductivity settings in Table 2 of the manuscript, where reentry is observed. The right panel of these movies corresponds to simulations with the faster conductivity settings, where no reentry is observed.

Movies S7-S14 correspond to the simulations in Figure 7 of the manuscript, which show changes in the membrane potential during pacing with the minimum S2 pacing CL. The left panel of these movies corresponds to simulations with the AF conductivity settings in Table 2 of the manuscript, where reentry is observed. The right panel of these movies corresponds to simulations with the faster conductivity settings, where no reentry is observed.

# Supplemental References


Arora, R., J. Ng, J. Ulphani, I. Mylonas, H. Subacius, G. Shade, D. Gordon, A. Morris, X. He, Y. Lu, R. Belin, J. J. Goldberger, and A. H. Kadish. 2007. "Unique autonomic profile of the pulmonary veins and posterior left atrium." *J Am Coll Cardiol* 49 (12):1340-8. doi: 10.1016/j.jacc.2006.10.075.

Bayer, J. D., R. C. Blake, G. Plank, and N. A. Trayanova. 2012. "A novel rule-based algorithm for assigning myocardial fiber orientation to computational heart models." *Ann Biomed Eng* 40 (10):2243-54. doi: 10.1007/s10439-012-0593-5.

Bayer, J. D., C. H. Roney, A. Pashaei, P. Jais, and E. J. Vigmond. 2016. "Novel Radiofrequency Ablation Strategies for Terminating Atrial Fibrillation in the Left Atrium: A Simulation Study." *Front Physiol* 7:108. doi: 10.3389/fphys.2016.00108.

Chevalier, P., A. Tabib, D. Meyronnet, L. Chalabreysse, L. Restier, V. Ludman, A. Alies, P. Adeleine, F. Thivolet, H. Burri, R. Loire, L. Francois, and L. Fanton. 2005. "Quantitative study of nerves of the human left atrium." *Heart Rhythm* 2 (5):518-22. doi: 10.1016/j.hrthm.2005.01.022.

Courtemanche, M., R. J. Ramirez, and S. Nattel. 1998. "Ionic mechanisms underlying human atrial action potential properties: insights from a mathematical model." *Am J Physiol* 275 (1 Pt 2):H301-21.

Farges, J. P., M. Ollagnier, and G. Faucon. 1977. "Influence of acetylcholine, isoproterenol, quinidine and ouabain on effective refractory periods of atrial and ventricular myocardium in the dog." *Arch Int Pharmacodyn Ther* 227 (2):206-19.

Kneller, J., R. Zou, E. J. Vigmond, Z. Wang, L. J. Leon, and S. Nattel. 2002. "Cholinergic atrial fibrillation in a computer model of a two-dimensional sheet of canine atrial cells with realistic ionic properties." *Circ Res* 90 (9):E73-87.

Liu, L., and S. Nattel. 1997. "Differing sympathetic and vagal effects on atrial fibrillation in dogs: role of refractoriness heterogeneity." *Am J Physiol* 273 (2 Pt 2):H805-16.

Molina, C. E., H. Gesser, A. Llach, L. Tort, and L. Hove-Madsen. 2007. "Modulation of membrane potential by an acetylcholine-activated potassium current in trout atrial myocytes." *Am J Physiol Regul Integr Comp Physiol* 292 (1):R388-95. doi: 10.1152/ajpregu.00499.2005.

Verkerk, A. O., G. S. Geuzebroek, M. W. Veldkamp, and R. Wilders. 2012. "Effects of acetylcholine and noradrenalin on action potentials of isolated rabbit sinoatrial and atrial myocytes." *Front Physiol* 3:174. doi: 10.3389/fphys.2012.00174.

Vigmond, E. J., V. Tsoi, S. Kuo, H. Arevalo, J. Kneller, S. Nattel, and N. Trayanova. 2004. "The effect of vagally induced dispersion of action potential duration on atrial arrhythmogenesis." *Heart Rhythm* 1 (3):334-44. doi: 10.1016/j.hrthm.2004.03.077.
