# Supplementary material for: Acetylcholine Delays Atrial Activation to Facilitate Atrial Fibrillation
Source: Front Physiol. 2019 Sep 4;10:1105. doi: 10.3389/fphys.2019.01105 (PMC6737394; doi:10.3389/fphys.2019.01105)
Supplement: Supplementary file 5 [file Presentation_4.PPTX]

## Slide 1
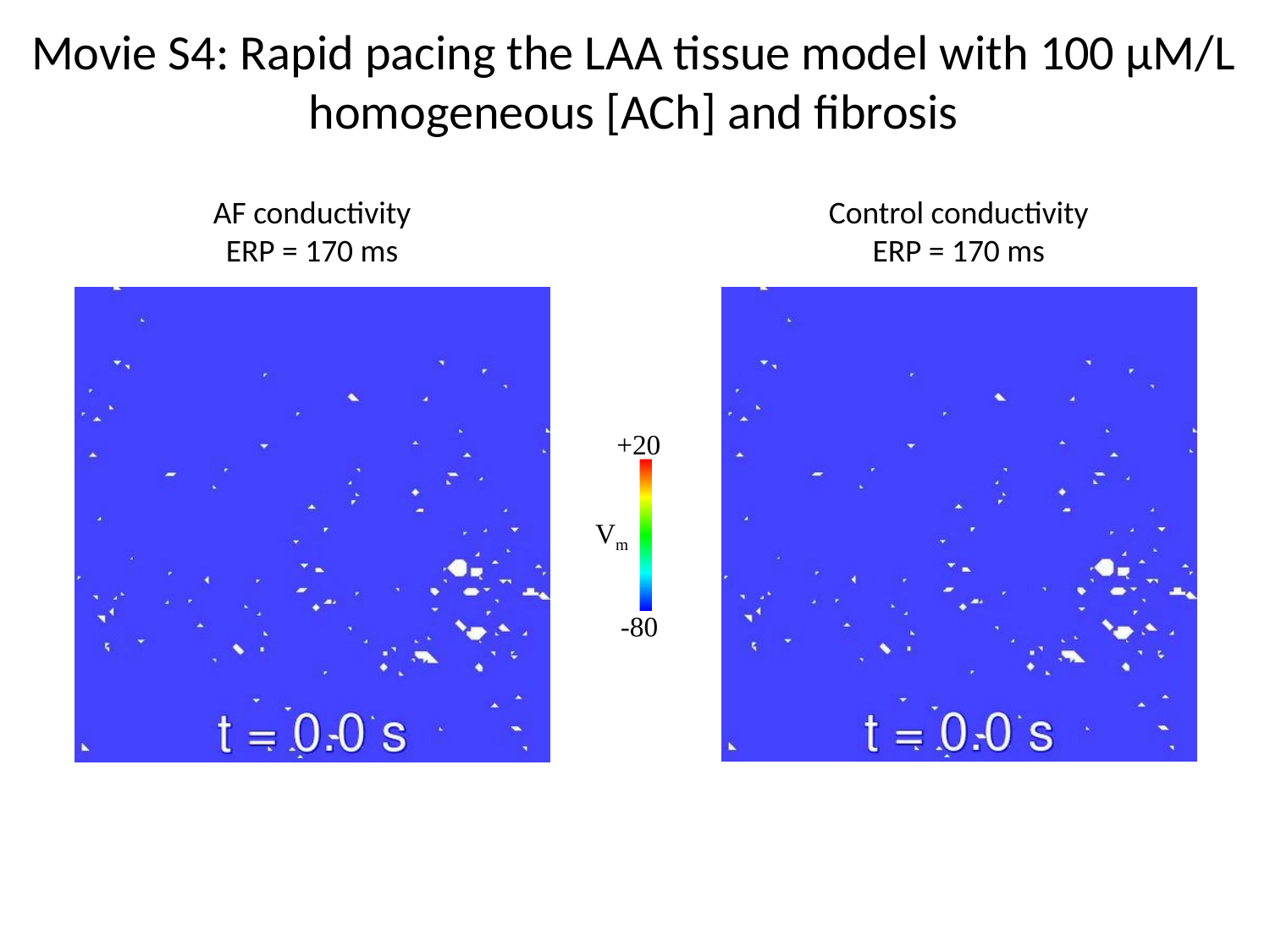

Movie S4: Rapid pacing the LAA tissue model with 100 µM/L homogeneous [ACh] and fibrosis
Control conductivity
ERP = 170 ms
AF conductivity
ERP = 170 ms
+20
Vm
-80
