# Supplementary material for: Acetylcholine Delays Atrial Activation to Facilitate Atrial Fibrillation
Source: Front Physiol. 2019 Sep 4;10:1105. doi: 10.3389/fphys.2019.01105 (PMC6737394; doi:10.3389/fphys.2019.01105)
Supplement: Supplementary file 11 [file Presentation_10.PPTX]

## Slide 1
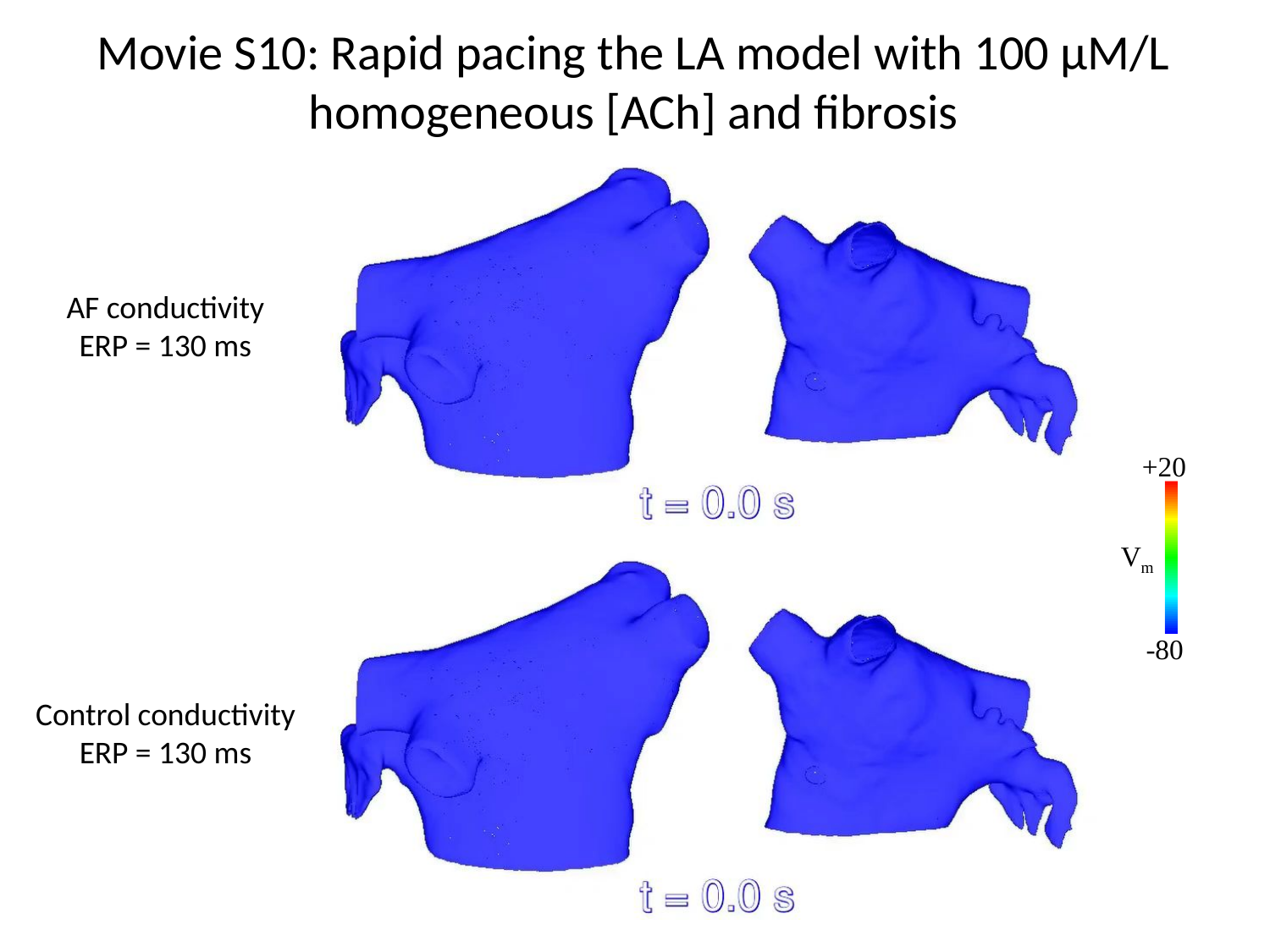

Movie S10: Rapid pacing the LA model with 100 µM/L homogeneous [ACh] and fibrosis
AF conductivity
ERP = 130 ms
+20
Vm
-80
Control conductivity
ERP = 130 ms
